# Distinct neural substrates of visuospatial and verbal-analytic reasoning as assessed by Raven’s Advanced Progressive Matrices

**DOI:** 10.1038/s41598-017-16437-8

**Published:** 2017-11-24

**Authors:** Zhencai Chen, Alain De Beuckelaer, Xu Wang, Jia Liu

**Affiliations:** 1 Department of Psychology, Jiangxi University of Traditional Chinese Medicine, Nanchang, China; 2 Institute for Management Research, Radboud University, Nijmegen, The Netherlands; 3 Department of Personnel Management, Work and Organizational Psychology, Ghent University, Ghent, Belgium; 4 School of Life Sciences, Beijing University of Chinese Medicine, Beijing, China; 5 Beijing Key Laboratory of Applied Experimental Psychology, School of Psychology, Beijing Normal University, Beijing, China

## Abstract

Recent studies revealed spontaneous neural activity to be associated with fluid intelligence (gF) which is commonly assessed by Raven’s Advanced Progressive Matrices, and embeds two types of reasoning: visuospatial and verbal-analytic reasoning. With resting-state fMRI data, using global brain connectivity (GBC) analysis which averages functional connectivity of a voxel in relation to all other voxels in the brain, distinct neural correlates of these two reasoning types were found. For visuospatial reasoning, negative correlations were observed in both the primary visual cortex (PVC) and the precuneus, and positive correlations were observed in the temporal lobe. For verbal-analytic reasoning, negative correlations were observed in the right inferior frontal gyrus (rIFG), dorsal anterior cingulate cortex and temporoparietal junction, and positive correlations were observed in the angular gyrus. Furthermore, an interaction between GBC value and type of reasoning was found in the PVC, rIFG and the temporal lobe. These findings suggest that visuospatial reasoning benefits more from elaborate perception to stimulus features, whereas verbal-analytic reasoning benefits more from feature integration and hypothesis testing. In sum, the present study offers, for different types of reasoning in gF, first empirical evidence of separate neural substrates in the resting brain.

## Introduction

Fluid intelligence (gF) is the ability to think abstractly and solve novel problems, independent of acquired knowledge^1,2^. Raven’s Advanced Progressive Matrices (RAPM) offers one of the most widely used nonverbal and culture-free tests in the study of gF^3,4^. RAPM quantifies individual differences in domain-general cognitive abilities such as perception, memory, and reasoning ability^5^. As such, RAPM includes multiple (item) subsets capturing distinctive aspects of gF-related cognitive processing.

Most studies conceptualize RAPM as a unidimensional test to primarily capture gF, and the full RAPM total score is used to rate individuals’ gF^6,7^. However, several studies (e.g., Carpenter *et al*., 1990; DeShon *et al*., 1995) have challenged this unidimensional conceptualization by revealing RAPM’s multidimensional structure, which is identified empirically through various techniques/paradigms such as the registration of eye-movements^3^, the verbal overshadowing paradigm^5^, and factor analysis of RAPM item scores^8,9^. For instance, Carpenter *et al*. (1990) subdivide all RAPM items into five distinct RAPM subsets and labeled them as “constant in a row”, “pairwise progression”, “addition or subtraction”, “distribution of three rules”, and “distribution of two rules”^3^. DeShon *et al*. (1995) identify two RAPM subsets and labeled them as the “visuospatial” and “verbal-analytic” subset^5^. Finally, Dillon *et al*. (1981) also form two, but different RAPM subsets, namely “pattern addition or subtraction” and “pattern progression”^8^.

Nevertheless, one should realize that, across alternative “subset structures” (e.g., across Carpenter *et al*.’s and DeShon *et al*.’ subsets), certain pairs of subsets may be substantially correlated due to a largely similar item composition of (correlated) subsets. For instance, all RAPM items assigned to Carpenter *et al*.’s “distribution of three” are also assigned to the “verbal-analytic” subset. Similarly, most items assigned to Carpenter’s “addition or subtraction” and “distribution of two rules” are also assigned to the “visuospatial” subset^5,10^. In sum, it is reasonable to subdivide RAPM items into a visuospatial and verbal-analytic subset: (1) visuospatial reasoning, which is overshadowed by verbal representation and involves dealing with figural problems requiring visual perception operations to graphical features (e.g., quantity, spatial location and direction) of RAPM items, and (2) verbal-analytic reasoning, which deals with analytic problem solving requiring logical operations to abstract attributes (e.g., category, distribution) of RAPM items^5,10,11^. Additional studies have attested to the two-dimensional RAPM structure by demonstrating that visuospatial reasoning, but not verbal-analytic reasoning, is correlated with spatial mental imagery and autism-related traits (e.g., Autism-Spectrum Quotient)^12–14^. The behavioral study of DeShon and colleagues (1995) clearly differentiates between visuospatial and verbal-analytic reasoning, but the study does not show whether or not dissociable neural substrates exist. The contemporary literature dealing with functional magnetic resonance imaging (fMRI) provides some preliminary results regarding dissociable neural substrates of gF as assessed by RAPM (including RAPM subsets).

To date, four existing task-state fMRI studies have explored separate neural substrates related to the different types of reasoning embedded within RAPM^11,14–16^. In a representative study by Prabhakaran and colleagues (1997)^11^, participants were in a scanner while completing RAPM items. This representative study showed that (1) both visuospatial and verbal-analytic reasoning activate working memory-related brain regions (e.g., right frontal region and bilateral parietal regions), and (2) in comparison to visuospatial reasoning, verbal-analytic reasoning activates additional verbal working memory and executive function regions in the left frontal lobe. However, neither this representative study nor the other three task-state fMRI studies^14–16^ took into account the following three considerations. First, verbal-analytic reasoning is harder in general, and thus required longer processing times on average than visuospatial reasoning^11^. Therefore, it is possible that higher neural activation of verbal-analytic reasoning may result from a higher cognitive load and a longer processing time^15^. Second, gF is conceived as a domain-general and integrated function^17,18^; therefore, communication among anatomically separated brain regions rather than local activation may better reflect gF^19,20^. Finally, the stimulus-driven factors mentioned above (i.e., item hardness / item processing time) may also impact the functional connectivity (FC) during task-state fMRI, because (1) fMRI signals in stimulus-driven recruitment of individual regions are modified by task conditions (e.g., differential item hardness) and then affect the FCs of these individual regions, and (2) to a certain extent the fluctuation of fMRI time course is stimulus-locked and determined by stimulus onset and duration of the stimulus that also affects FC during the task-state^21,22^.

To adequately deal with these considerations our present study explores global brain (functional) connectivity (GBC) during resting-state fMRI (rs-fMRI) to identify the neural substrates of the two types of reasoning embedded within RAPM. In this exploration we measure spontaneous neural activity of a subject’s brain while the subject is ‘in rest’, that is not being occupied with processing emerging from completing a specific task; being in rest helps avoiding unwanted impact (i.e., bias in results) due to differences between the different types of stimuli (e.g., the more cognitive load and longer processing time required for verbal-analytic RAPM items). Besides, functional connectivity (FC) helps quantifying the extent to which multiple regions are working as a cohort, and enables the identification of neural correlates of gF at network level. Once FC is expressed the GBC value is be calculated. The GBC value is defined as the average FC of a voxel to all other voxels in the brain. As GBC values reflect the global connectivity of brain voxels our present study thus explores the neural correlates of gF at a global and interactive level, and distinguishes itself from previous fMRI studies which examined local properties of certain regions^17^. By exploring the relation between the GBC value of a voxel to the behavioral performance measured for visuospatial (i.e., relation 1) and verbal-analytic reasoning (i.e., relation 2) and making systematic comparisons between these relations, one may identify neural substrates underlying these two types of reasoning embedded within RAPM.

To date the Parieto-Frontal Integration Theory (P-FIT) constitutes one of the most widely accepted theories on the neural substrates of intelligence. The P-FIT conceives intelligence as the product of an interaction involving distributed brain regions mainly in frontal and parietal cortices and visual and temporal lobes^18,19^. Based on the P-FIT we hypothesized that visuospatial reasoning is likely to be related to the visual cortex and, in contrast to visuospatial reasoning, verbal-analytic reasoning relies more heavily on cortical regions involved in feature integration (e.g., the angular gyrus)^20^, because visuospatial reasoning requires processes of visual perception whereas verbal-analytic reasoning depends more on operating abstract rules which initiate from integrating features (e.g., shape-spatial-numeric associations)^5,10,11^.

## Results

### Behavioral statistics

All descriptive statistics (e.g., skewness, kurtosis and score range) pertaining to the full RAPM total score, the visuospatial and the verbal-analytic subset scores are displayed in Table 1. A Kolmogorov-Smirnov (univariate) normality test produced a non-significant *p*-value (i.e., asymptotic significance *p* = 0.14), implying a normally distributed full RAPM total score. In contrast, the visuospatial and verbal-analytic subset scores followed a non-normal distribution (i.e., asymptotic significance *ps* < 0.001). Cronbach’s alpha values showed a moderate internal consistency for the full RAPM total score and low to moderate internal consistencies for the subset total scores (see Table 1). Besides, the correlation coefficient between the visuospatial and verbal–analytic subset total scores was 0.43, indicating a moderate correlation. The moderate correlation indicates that the covariation between both types of reasoning’s subsets could only explain 18% (i.e., 0.18 = 0.43 squared) of the total variation in each type of reasoning’s subset, leaving 82% of the total variation as unique and random variation. The unique variation of each particular type of reasoning’s subset may help identifying differences in the mechanisms underlying visuospatial and verbal-analytic reasoning. Besides, consistent with previous studies^11,15^, the participants considered the items of the verbal-analytic subset more difficult than those of the visuospatial subset (*t*_[259]_ = 21.71, *p* < 0.001).

**Table 1:** Participants’ descriptive statistics for full RAPM item set, visuospatial and verbal-analytic items.

Items	Full (set of) RAPM items (36 items)	Visuospatial items (13 items)	Verbal-analytic items (12 items)
Mean total score (M)	26.23	10.53	7.44
Standard deviation (SD)	3.70	1.43	1.80
Skewness (SK)	0.04	−0.46	0.12
Kurtosis (KU)	−0.60	0.19	−0.31
Minimum score (MIN)	18	6	3
Maximum score (MAX)	35	13	12
Cronbach’s alpha	0.69	0.35	0.48

### Correlations between visuospatial, verbal–analytic scores and GBC value

**Figure 1:**
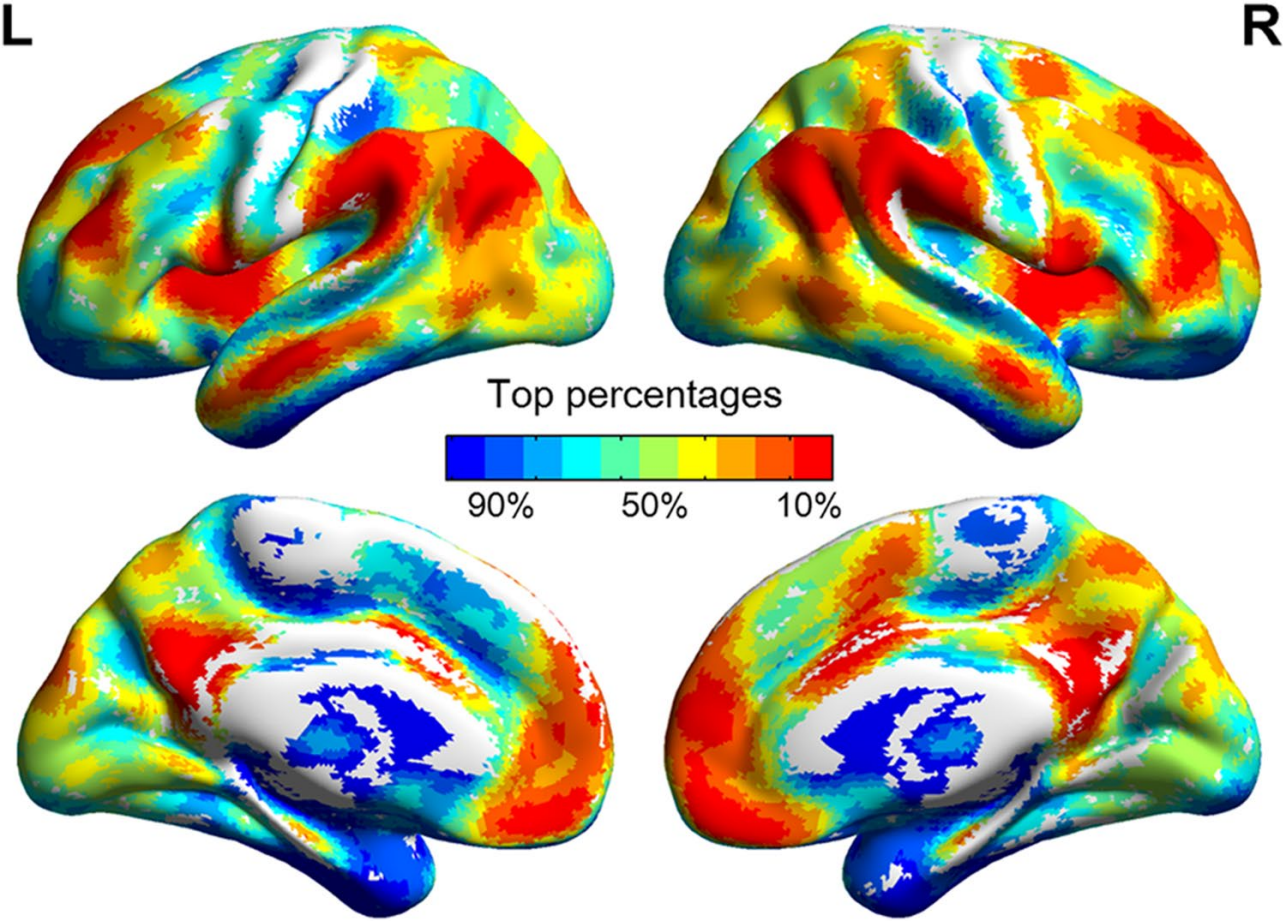
Mean GBC values averaged across participants by top percentages of voxels. High GBC values are shown in warm colors (red), low GBC values are shown in cool colors (blue).

**Figure 2:**
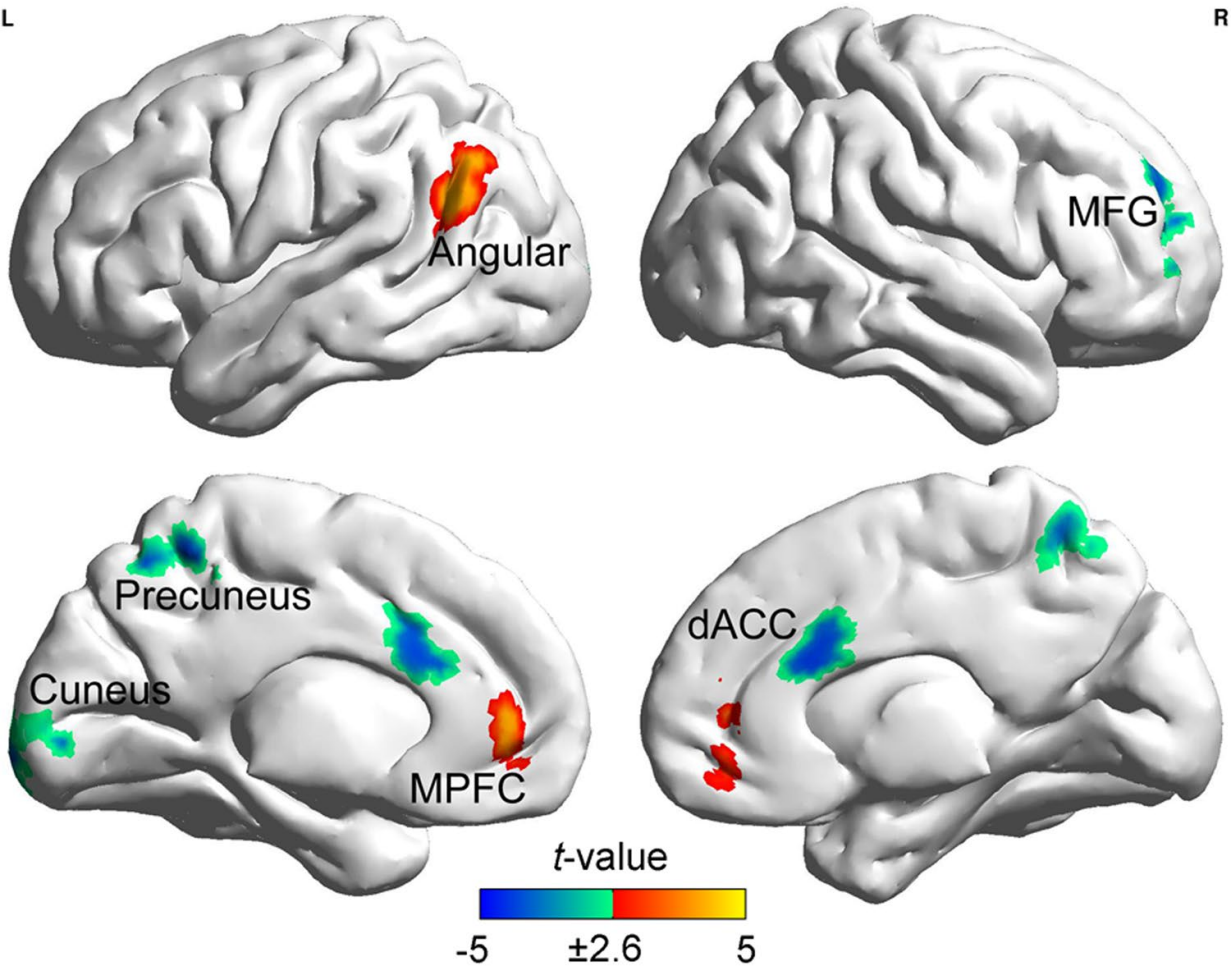
Regions with GBC values showing significant correlations with the RAPM score after the effects of sex, age and FD scores were regressed out. Clusters containing more than 122 contiguous voxels survived the AlphaSim correction. Abbreviations: MFG, middle frontal gyrus; MPFC, medial prefrontal cortex; dACC, dorsal anterior cingulate cortex.

In Fig. 1, the mean GBC image showed a spatial intensity distribution similar to those of previous studies^21^, that is the multiple demand regions (MDr) and default mode network (DMN) regions displayed highest GBC in the brain. The size of the correlation as computed between the total RAPM scores and the GBC values pointed toward anterior brain regions in the middle frontal gyrus (MFG), medial prefrontal cortex (MPFC), and dorsal anterior cingulate cortex (dACC), as well as the posterior brain regions in the angular, supramarginal gyrus, precuneus, and cuneus (Fig. 2 and Table 2). Those regions, presently identified with spontaneous FCs, correspond with characteristic regions of P-FIT, suggesting that these regions may serve as the neural substrates of gF^18^.

**Table 2:** Regions with GBC values showing significant correlations with the full RAPM score.

Region	Brodmann area	No. of voxels	Peak *t* (*r*)-value	x	y	z
R. Middle/ Superior frontal gyrus	10	206	−4.22 (−0.25)	35	50	22
Medial frontal gyrus	32	180	3.59 (0.22)	0	48	−6
Dorsal anterior cingulate cortex	24/32	288	−4.03 (−0.24)	4	20	24
Precuneus	7	172	−3.99 (−0.24)	2	−52	58
L. Angular/ Supramarginal gyrus	39	285	3.79 (0.23)	−54	−60	26
Cuneus/ Lingual gyrus	17/18	122	−3.62 (−0.22)	−6	−102	−6

**Figure 3:**
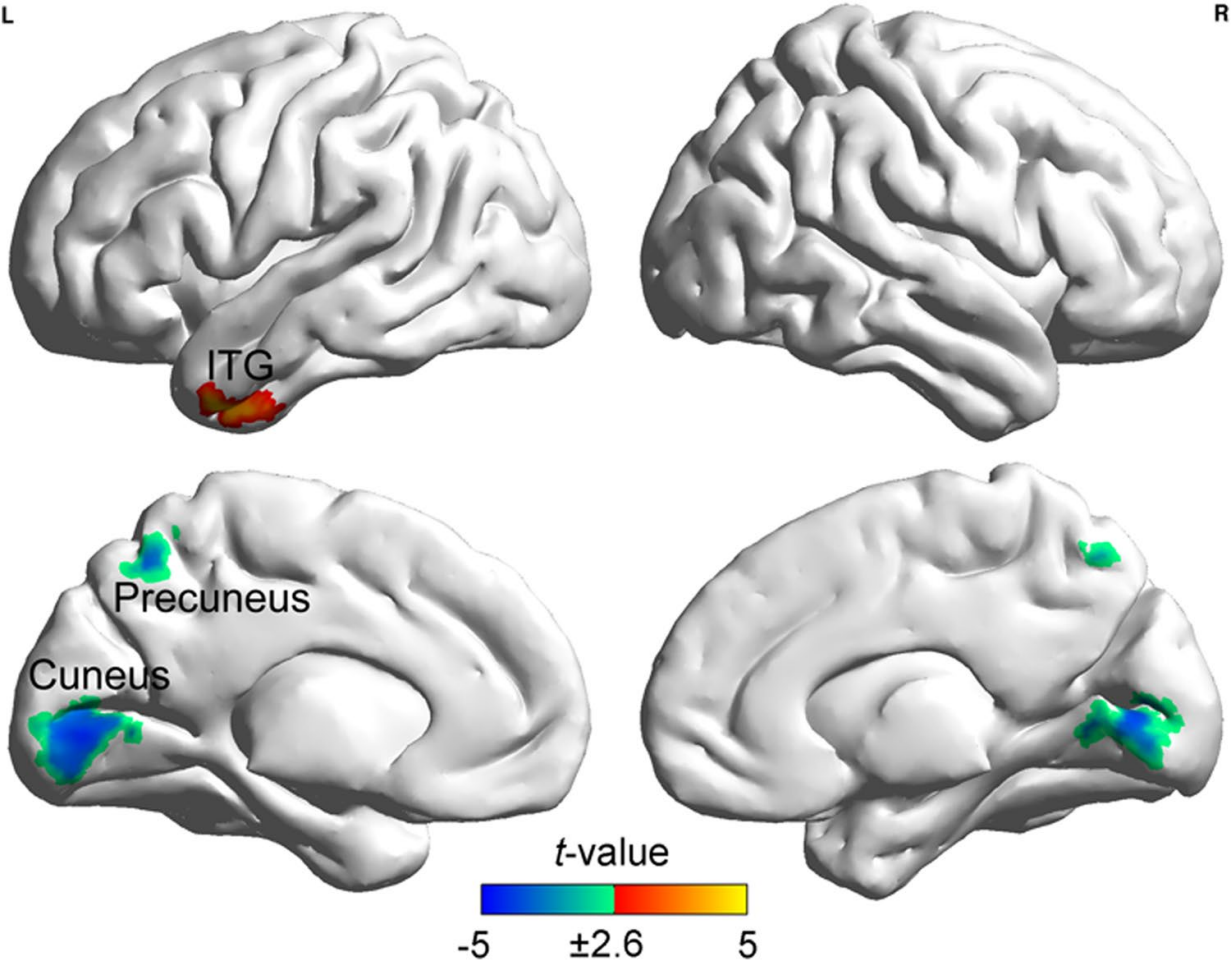
Regions with GBC values that were significantly correlated with the visuospatial subset score after regressing out the following covariates: sex, age, FD and the verbal–analytic subset score. Clusters containing more than 116 contiguous voxels survived the AlphaSim correction. Abbreviation: ITG, inferior temporal gyrus.

To identify GBC patterns of visuospatial reasoning, the correlation between the GBC value and the visuospatial subset score was computed (Fig. 3 and Table 3). The results show that the functional connectivities (FCs) of GM voxels in the left middle and inferior temporal gyri (MTG, ITG) positively correlate with the visuospatial subset score, whereas the FCs of GM voxels in the primary visual cortex (PVC) and the precuneus negatively correlate with the visuospatial subset score.

**Table 3:** Regions with GBC values showing significant correlations with the visuospatial and the verbal-analytic subset score.

Region	Brodmann area	No. of voxels	Peak *t* (*r*)-value	x	y	z
**Visuospatial subset**						
L. Middle/ Inferior temporal gyrus	21/20	159	4.57 (0.27)	−46	8	−38
Precuneus	7	120	−4.47 (−0.27)	0	−60	56
Cuneus/ Lingual gyrus	18/17	617	−4.29 (−0.26)	0	−80	0
**Verbal-analytic subset**						
R. Inferior frontal gyrus/ Insula	47/13	265	−4.78 (−0.29)	44	28	0
Dorsal anterior cingulate cortex	24	138	−3.99 (−0.24)	4	18	28
Supplementary motor area	6	148	−4.01 (−0.24)	22	8	62
L. Angular gyrus	39	115	4.19 (0.25)	−46	−60	24
R. Angular/ Supramarginal gyrus	39	163	4.03 (0.24)	52	−54	24
R. Temporo-parietal junction	40	110	−3.46 (−0.21)	60	−34	38

**Figure 4:**
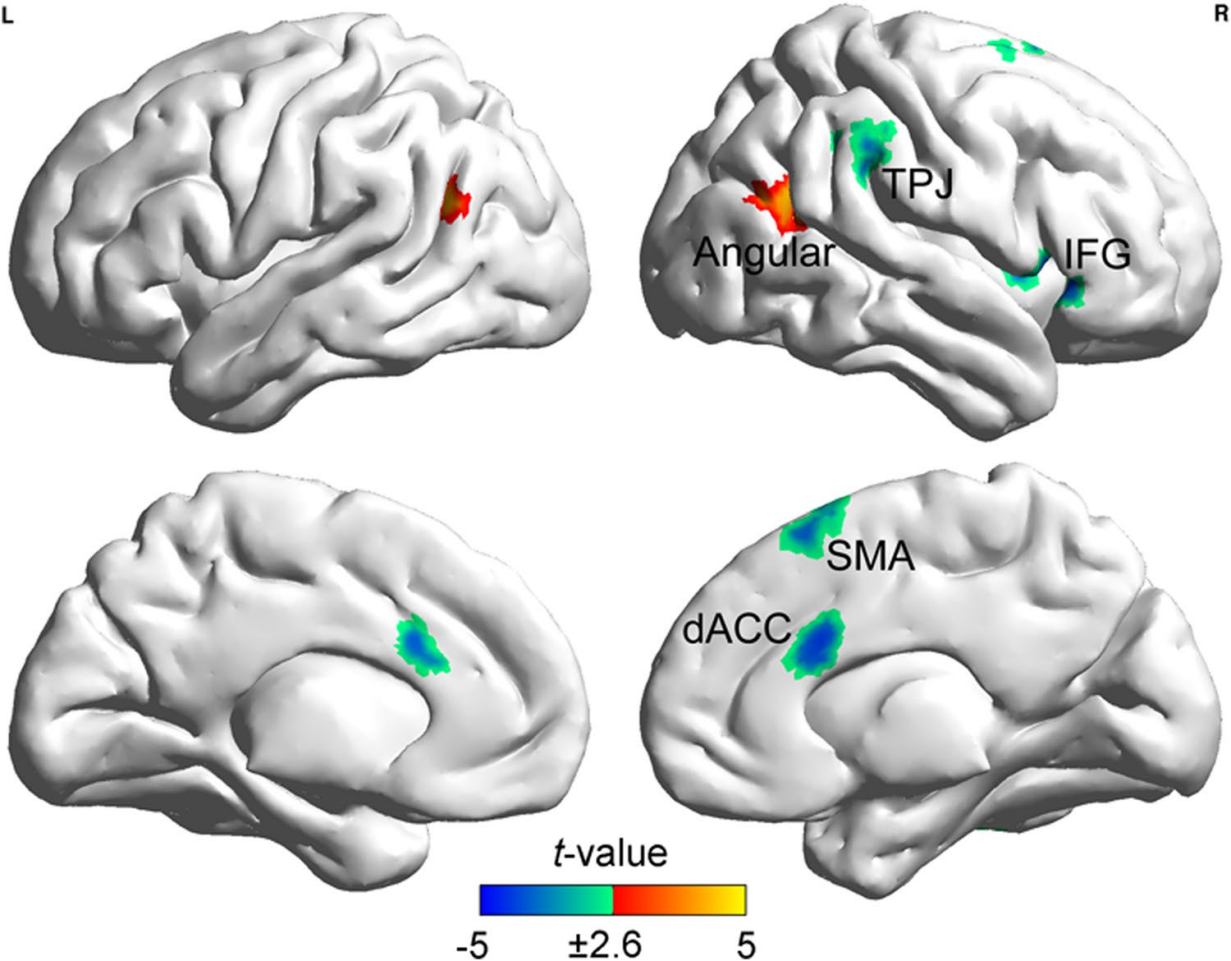
Correlational patterns between the GBC value and the verbal–analytic subset score after the effects of sex, age, FD and the visuospatial subset score were regressed out. Clusters containing more than 105 contiguous voxels survived the AlphaSim correction. Abbreviations: IFG, inferior frontal gyrus; dACC, dorsal anterior cingulate cortex; SMA, supplementary motor area; TPJ, temporoparietal junction; ITG, inferior temporal gyrus.

GBC patterns of verbal-analytic reasoning as depicted by the correlation between the GBC value and the verbal–analytic subset score are shown in Fig. 4 and Table 3. The correlational pattern reveals that FCs of GM voxels in the angular gyri of both sides positively correlate with the verbal–analytic subset score, and the FCs of GM voxels in the right inferior frontal gyrus (rIFG), dACC, right temporoparietal junction (TPJ), and the supplementary motor area (SMA) negatively correlate with the verbal–analytic subset score. The different relationships with the GBC value, as reflected in distinct correlational patterns for the two RAPM subsets, point toward different reasoning strategies underlying these RAPM subsets.

### Functional discriminations between visuospatial and verbal–analytic reasoning

**Figure 5:**
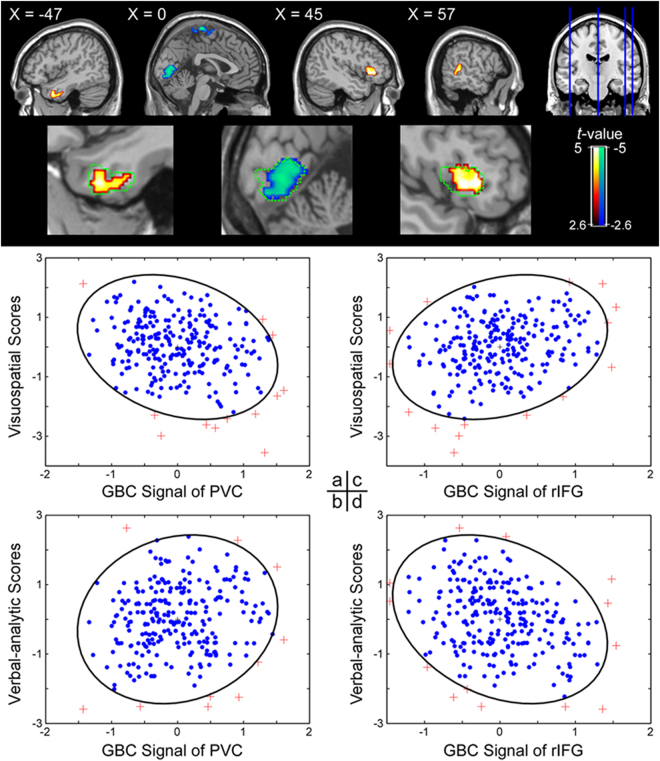
The upper panel depicts the interaction regions between visuospatial and verbal-analytic reasoning. Clusters containing more than 116 contiguous voxels survived the AlphaSim correction. The green dotted line shows the brain-behavioral GBC correlation regions of visuospatial and verbal-analytic reasoning; these correlation regions partially overlap; that is, they show a significant brain-behavioral correlation and, at the same time, a significant interaction of visuospatial and verbal-analytic reasoning. The bottom panel depicts correlational patterns between: (**a**) the primary visual cortex’s (PVC) GBC value and the visuospatial subset score; (**b**) the PVC’s GBC value and the verbal–analytic subset score; (**c**) the right inferior frontal gyrus’s (rIFG) GBC value and the visuospatial subset score; and (**d**) the rIFG’s GBC value and the verbal–analytic subset score. These visuospatial and verbal-analytic reasoning scores were centered using z-transform. The elliptic boundary defines the 95% confidence region.

To further explore the distinct neural substrates of visuospatial and verbal–analytic reasoning “interaction regions” were determined. More specifically, the difference (or “contrast”) between the visuospatial and verbal–analytic regression slopes were computed (when relating to the GBC value). Interaction regions are defined as regions for which the correlations for visuospatial reasoning and verbal-analytic reasoning with the GBC value are significantly different in the regression slopes. These interaction regions included the rIFG, SMA, PVC, left MTG/ ITG and right MTG (the upper panel of Fig. 5 and Table 4); note that some interaction regions (i.e., rIFG, PVC and left MTG/ ITG) partially overlap (see green dotted line in upper panel of Fig. 5) with the regions that were found to have significant associations between the GBC values and gF subsets in the separate analyses presented above (Figs 3 and 4). As opposed to non-overlapping regions the overlapping regions may more solidly discriminate between visuospatial and verbal–analytic reasoning.

**Table 4:** Cerebral regions reflecting the interaction between visuospatial and verbal-analytic reasoning.

Region	Brodmann area	No. of voxels	Peak *t* (*r*)-value	x	y	z
R. Inferior frontal gyrus	47/45	206	4.88 (0.29)	44	28	0
Medial frontal gyrus/ Paracentral lobule	6/4	159	−4.48 (−0.27)	0	−26	72
L. Middle/ Inferior temporal gyrus	21	120	4.27 (0.26)	−48	10	−38
R. Middle temporal gyrus	22	146	3.97 (0.24)	68	−40	4
Cuneus/ Lingual gyrus	18/17	479	−4.03 (−0.24)	6	−76	8

To obtain useful displays the region of interest (ROI) signal values were further extracted in the stimulus input level PVC (peak coordinate: 0, −80, 0) and the higher cognitive level rIFG (peak coordinate: 44, 28, 0). Scatterplots depicting the ROI signal values and the subset scores indicated that the visuospatial subset score is negatively correlated with the GBC value of the PVC (Fig. 5a), whereas the verbal–analytic subset score is positively correlated with the GBC value of the PVC (Fig. 5b). Oppositely, the visuospatial subset score is positively correlated with the GBC value of the rIFG (Fig. 5c), whereas the verbal–analytic subset score is negatively correlated with the GBC value of the rIFG (Fig. 5d). These results (describing interactions) further attest to the existence of different reasoning strategies underlying both RAPM subsets. In addition, the brain-behavioral GBC interactions for other visuospatial and verbal–analytic reasoning interaction regions are displayed in Figure S5 in the supplementary study materials.

**Figure 6:**
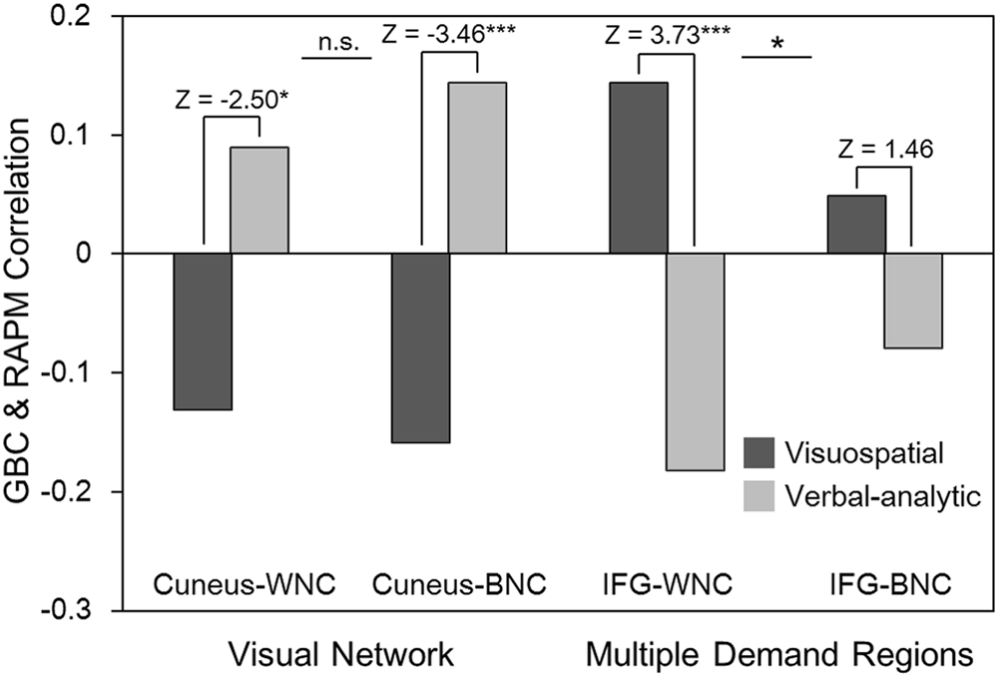
The visuospatial and the verbal-analytic scores correlate with both the within- and between- network GBC (WNC/ BNC GBC) of primary visual cortex (Cuneus) and inferior frontal gyrus (IFG) in the visual network and frontal-parietal multiple demand regions (i.e., the frontal-parietal network, dorsal attention network and ventral attention network)^71^.

**Figure 7:**
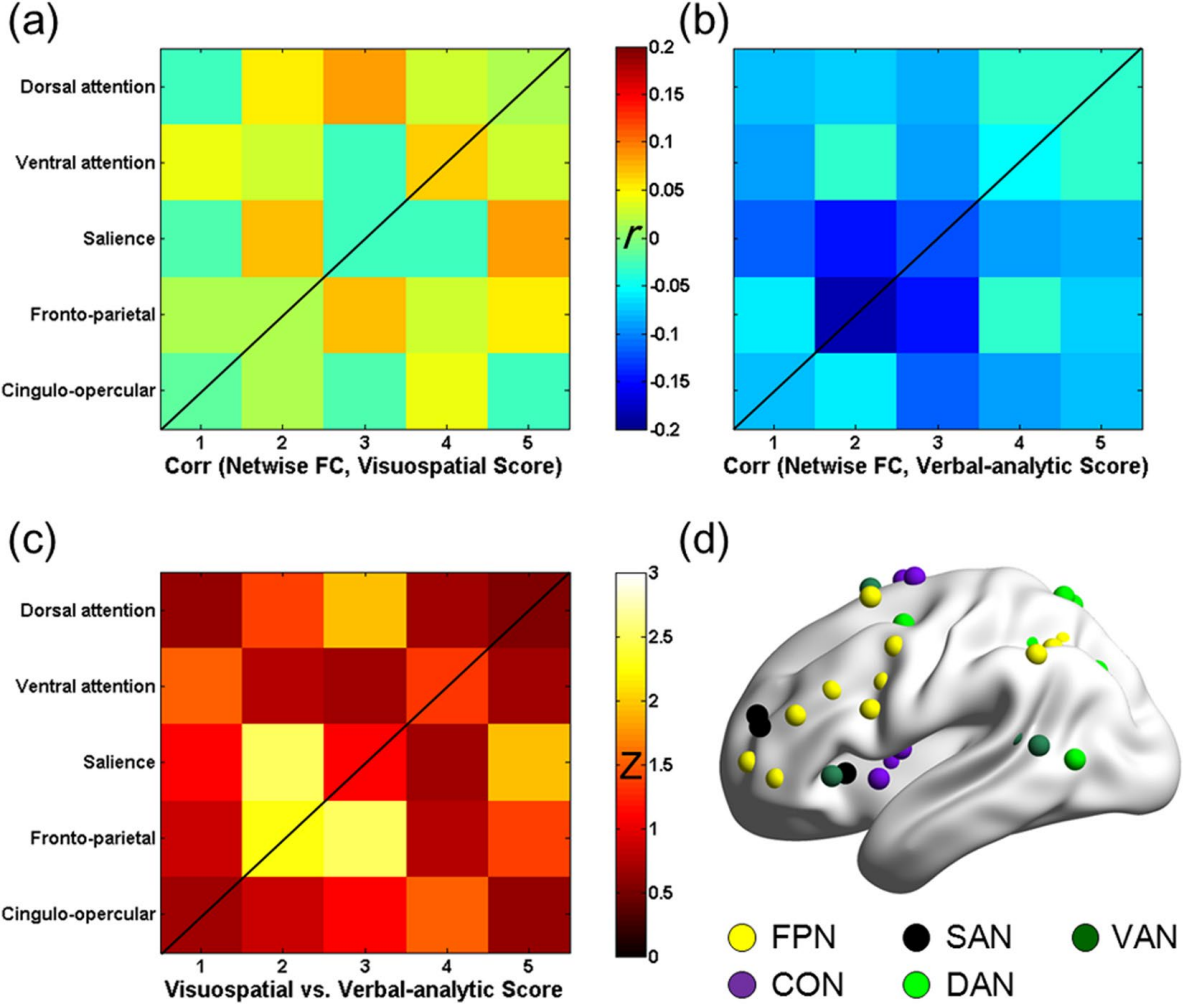
The correlations between the RAPM subset scores and the network-wise FCs in frontal and parietal regions. (**a**) The network-wise correlations of visuospatial and (**b**) verbal-analytic subsets; (**c**) the network-wise correlation differences between the visuospatial and the verbal-analytic subsets: the FCs within the frontal-parietal network (FPN) and the FCs between FPN and salience network (SAN) have significant different network-wise correlations with the two RAPM subset scores (false discovery rate corrected, *p* < 0.05); (**d**) these analyses pertain to the frontal and parietal multiple demand regions which includes the FPN, cingulo-opercular network (CON), SAN, dorsal attention network (DAN) and ventral attention network (VAN)^72^.

In addition to the whole brain GBC analyses, further understanding of the distinct neural mechanisms underlying visuospatial and verbal-analytic reasoning may emerge from the following analyses: the within- and between- network GBC (WNC / BNC GBC) analyses showed that both the WNC GBC and BNC GBC of visual cortex almost equally contributed to the neural distinction between the visuospatial and the verbal-analytic subsets. In contrast, the WNC/ BNC GBC analyses of MDr showed that the WNC GBC contributed more dominantly (i.e., much stronger) to the distinction between the two subsets (Fig. 6). Furthermore, the mean within-network connectivity (mWNC, e.g., the mean FCs between all frontal-parietal network [FPN] nodes) and mean between-network connectivity (mBNC, e.g., averaging all FCs between each couple of FPN and salience network [SAN] nodes) analyses showed that the FCs within FPN and the FCs between FPN and SAN contribute most significantly to the neural distinction between the visuospatial and the verbal-analytic subset (Fig. 7).

## Discussion

Our present study provides rs-fMRI data and data on a pencil-and-paper RAPM test to empirically validate the idea of distinct neural bases underlying visuospatial and verbal–analytic reasoning. Consistent with the P-FIT (Jung & Haier, 2007) our brain-behavioral correlations show that: (1) the RAPM score correlates with perception, hypothesis testing and cognitive control (e.g., attention and response selection during cognitive processing) regions, (2) the visuospatial subset score correlates with feature perception regions, (3) the verbal–analytic subset score correlates with feature integration, hypothesis testing and cognitive control regions, and (4) the data provide support for the functional discriminations between the two kinds of reasoning in the resting-state functional connectivity of the P-FIT-related brain regions (e.g., PVC and the rIFG).

During the resting-state, the regions that show a significant brain-behavioral correlation between the total RAPM score and the positive GBC value fit nicely with the four-stage model of the P-FIT: sensory information processing (e.g., cuneus, lingual gyrus and precuneus), integration and abstraction of sensory information (e.g., angular and supramarginal gyrus), hypothesis testing to find a solution for a problem (e.g., MFG), and response selection when the best solution is determined in hypothesis testing (e.g., dACC)^18,19^. Besides, the GBC value of the regions in the DMN, such as the MFG and angular gyrus, is found to be positively associated to the full RAPM score, suggesting that the increased DMN integration during the resting-state may benefit gF^22,23^. Our present study also reveals an association of the MFG and dACC with gF. Previous studies have shown that the MFG and dACC play an important role in the higher-level cognitive processes (when reasoning): relational integration, working memory, and cognitive control^11,15,16^; therefore, our findings also suggest a possible link between these high-level cognitive processes and gF.

Furthermore, the sign of the brain-behavioral correlations identified may reflect functional segregation (i.e., strengthened interregional connections) or integration (i.e., weakened interregional connections) of a certain region’s communication with other brain regions benefitting gF. As one of major organization principles of the human brain^24,25^, the mechanisms of integration and segregation are important for gating information flow^26–28^. Another major organization principle of the human brain is the anticorrelation between the DMN and task-positive network (TPN). Both networks show different activity and FC patterns that depend on brain states (e.g., resting vs. task-state). The DMN likely has higher activity and FC strengths than the TPN during the resting-state and vice versa during the task-state^29^. In addition, the abnormal activity and FC strengths (e.g., the decreased FC of the DMN during resting-state) may relate to impaired or lower-level cognitive abilities (e.g., undergoing brain diseases and aging)^30,31^. According to these major brain organization principles, one may conclude that, during the resting-state, in some DMN regions (e.g., MPFC and angular gyrus) functional integration may benefit the gF reasoning while in some TPN regions (e.g., MFG and dACC) functional segregation may benefit the gF reasoning.

In line with our results, previous FC studies, which were typically based on seed-based analyses^30^, small world attribute analyses^31^, GBC analyses of inter-task resting blocks^32^, and resting-state fMRI^33^ also identified cortical regions in the frontal and parietal cortexes that are associated with gF. However, our study, as well as Hilger and colleagues’s (2017), both of which relied on GBC analyses of “pure” resting-state fMRI, failed to identify the dorsal lateral prefrontal cortex (DLPFC) observed in Cole *et al*.’s study (2012). Such difference may reflect the fact that interval resting blocks between N-back task blocks were used in Cole *et al*.’s analysis (2012). Although the fMRI signals of the intervals were careful controlled for from task conditions, participants were not at rest during the interval because they might have prepared for the incoming task blocks of the “N-back” task^34,35^.

### Visuospatial reasoning

Our present study shows that the PVC and the precuneus’s GBC value are negatively correlated to the visuospatial subset score. Namely, the more strongly the PVC and the precuneus are connected to other brain regions during the resting-state, the more errors participants make while completing the visuospatial items. In prior studies, it was found that: (1) the stimuli used to assess visuospatial reasoning are easier to envisage visually than the stimuli used to assess verbal–analytic reasoning^11,36^, (2) the PVC and precuneus separately relate to functions of primary stimulus perception and visuospatial imagery^37,38^. Thus, the gF-beneficial low-level GBC of the PVC and precuneus during resting-state may relate with an elaborate stimulus feature processing and selection in visuospatial reasoning^14^.

### Verbal-analytic reasoning

According to the P-FIT^18,19^: (1) the angular and supramarginal gyrus respond to the integration and abstracting of sensory information, (2) the IFG tends to attend to hypothesis testing and cognitive control to find a solution for a given problem, and (3) the dACC is related to response selection whenever the best solution is determined in hypothesis testing. In our present study we observe a positive correlation between the verbal-analytic subset score and the angular gyrus’ GBC value during the resting-state. As the angular gyrus integrates and reorients attention to task-relevant information^18–20^, this positive correlation indicates that the verbal–analytic items tend to rely on the feature integration function of the angular gyrus to organize the more complicated verbal–analytic item stimulus features and rules (e.g., rules from the integrated shape, spatial and numeric RAPM features)^5,39^. For the other verbal–analytic item-related regions, previous studies also indicated that the rIFG, the dACC, and the TPJ may involve in the hypothesis testing and cognitive control functions^18,19,40,41^ as well as the attention to salient stimulus^42,43^. Our present study reveals that the rIFG, the dACC, and the TPJ’s GBC value is ne*g*atively related to the verbal-analytic subset score, which indicates that individuals pay more effort to endogenously generate and maintain several hypotheses and goals (e.g., the candidate rules and steps) when responding adequately to verbal–analytic items than to visuospatial items^3,44^. Thus, according to results produced by the resting-state brain, verbal–analytic reasoning may benefit from the stimulus feature integration, top-down cognitive control and hypothesis testing.

The interactions found between visuospatial and verbal-analytic reasoning in the rIFG, SMA, PVC, left MTG/ ITG and right MTG further attest to the cerebral functional dissociation of the two gF subsets. However, the dACC, precuneus and angular gyrus’s GBC which were found to only display significant correlations with one subset (i.e., visuospatial or verbal-analytic reasoning) do not display significant interactions between the subsets. This indicates that, although not both subsets revealed significant correlations with the GBC of the dACC, precuneus and angular gyrus, both subsets may have some relationships with these regions’ GBC. Besides, the interaction regions in the SMA and right MTG show a non-significant correlation between the subset score and the GBC value in separate analyses of the visuospatial and verbal-analytic subsets. These regions (SMA, right MTG) should be cautiously regarded when considering their roles in distinguishing neural mechanisms underlying visuospatial and verbal-analytic reasoning. Unlike the dACC, precuneus, angular gyrus, SMA and right MTG, the interaction regions in the left MTG/ ITG, PVC and rIFG partially overlap with the regions that have significant correlations between the GBC values and one of the gF subsets. As such, as indicated by different brain-behavioral correlation directions with RAPM subsets, it is possible that a cognitive function that benefits one type of gF reasoning may hinder the other type of gF reasoning. Specifically, the bottom-up elaborate representation of visual feature in PVC facilitates visuospatial reasoning but it may hinder verbal-analytic reasoning. Analogously, the endogenously attention and hypothesis testing of rIFG may benefit verbal-analytic reasoning but hinder the exogenously feature perception of visuospatial reasoning.

Taken together, during the resting-state, distinct neural substrates for visuospatial and verbal-analytic reasoning manifest themselves in the following way: these two types of reasoning appear to be separately associated with the functional connectivity of two distinctive sets of distributed brain regions, namely the functional connectivity of PVC for visuospatial reasoning, and the functional connectivity of rIFG for verbal-analytic reasoning. This distinction in cerebral functional integration goes hand in hand with two distinct representational codes for each RAPM item (i.e., visual and propositional representations) and –as such – is informative with respect to the cognitive mechanisms underlying the distinct neural substrates for visuospatial and verbal-analytic reasoning. Specifically, the visual and propositional representations are not equally spread across visuospatial and verbal-analytic items, and this unequal spread may lead to distinct reasoning types for different RAPM items. For instance, the visual representation of a visuospatial item is more accessible to working memory than the propositional representation, thereby rendering the less accessible (or weaker) propositional representation ineffective, and verbal-analytic reasoning less likely to be used during the processing of a visuospatial item^5^.

Furthermore, the WNC/BNC GBC results of visual cortex further indicate that the segregation and integration of visual features for visuospatial and verbal-analytic subsets have distinct underlying neural mechanisms which are manifested in different FC patterns within the visual cortex and different FC patterns between the visual cortex and the high-level brain regions. As far as MDr is concerned, unlike the BNC GBC, the WNC GBC plays a dominant role in distinguishing visuospatial and verbal-analytic subsets, a finding that is consistent with Woolgar and colleagues (2010) who concluded that gF is more linked to FCs within the MDr than to FCs between MDr and other brain regions. And the network-wise brain-behavioral correlations in Fig. 7 further show that both the FCs within FPN and the FCs between FPN and SAN contributed most significantly to the neural distinction between visuospatial and verbal-analytic subsets in MDr. Thus, the FPN and SAN may be gF-related core networks in MDr. In short, these findings which offer more detailed descriptions of gF-related visual network and MDr functional connectivity contribute to a further understanding of the distinct neural mechanisms underlying visuospatial and verbal-analytic reasoning.

The dissociable neural correlates of the two subsets of RAPM reinforces the idea that RAPM has a multidimensional rather than a unidimensional structure, and therefore, a replacement of the full RAPM total score (i.e., one predictor) with multiple RAPM dimension scores (i.e., multiple predictors) helps in gaining accuracy when it comes to predicting individual differences in the gF (e.g., perceptual and analytic abilities). For example, if two individuals are obtained the same full RAPM total score (a situation that is commonly encountered in real tests)^45^, with a unidimensional view on gF, one may logically conclude that both individuals are identical in terms of gF. In contrast, with a multidimensional view on gF, despite their identical full RAPM total score, one may still discover that both individuals differ in perceptual and analytic abilities.

At the cerebral level, our results fit nicely with behavioral results of DeShon and colleagues’ (1995), a study which aimed at differentiating between the visuospatial and verbal–analytic items included in RAPM. Our present study further extends their behavioral results by showing dissociable resting-state neural substrates of two distinct RAPM subsets. Consequently, as already touched upon above, the practice of working with two unique (RAPM) performance scores (rather than just one full RAPM performance score) should be thought of as being ‘superior’, especially if one aims at getting a full appreciation of RAPM’s components as well as gF. Follow-up research may attempt to separate the brain mechanisms of RAPM’s subsets with other classifications (e.g., the more complex RAPM subsets of Carpenter *et al*. (1990)) and produce standards (e.g., using normative groups) to better interpret individuals’ subset scores as computed in the RAPM test.

## Methods

### Participants

Two hundred and sixty-six healthy college students (146 females, mean age in sample is 21.2 years; standard deviation (SD) is 1.1 years; range is 18.4–24.8 years) were recruited for the RAPM test and MRI scanning (i.e., rs-fMRI and structural MRI). All participants were right-handed, implying that a potential “handedness effect” on brain organization was avoided. The data from six participants were not analyzed due to head motions exceeding 2.0° in rotation or 2.0 mm in translation. The collection of MRI data for our present study was part of a larger project that aimed at exploring the neural correlates of human cognition under the interaction between ‘nature’ and ‘nurture’^46–50^. All MRI studies related to this larger project were approved by the Institutional Review Board of Beijing Normal University (BNU), Beijing, China, and written consent was obtained from each participant.

### Structure of RAPM and subset score computation

Conventionally, RAPM is composed of 48 nonverbal picture items, shortly referred to as ‘items’. The 48 items can be subdivided in one set of 12 items (set I) and another set of 36 items (set II)^4^. During a pencil-and-paper RAPM test, participants were instructed to identify, for each item, the missing piece of a 3 × 3 × 3 picture matrix out of eight candidate pictures. The 12 items in set I were used to practice with the instructions given. Then the 36 items in set II had to be completed within 30 minutes so that participants’ visuospatial and verbal-analytic subset scores could be derived. Items in set II were further classified in accordance with DeShon’s subsets. More specifically, items 3, 7, 9, 10, 11, 12, 16, 18, 22, 23, 24, 32, and 33 compose a subset of 13 visuospatial items, and items 1, 4, 8, 13, 17, 21, 27, 28, 29, 30, 34, and 36 compose a subset of 12 verbal-analytic items. The remaining items (11 in total) do not belong to either subset^5^, but their scores are needed to compute the full RAPM total score (i.e., the total sum score based on all set II items). Both the full RAPM total score as well as the subset total (and mean) scores were derived based on correct or incorrect processing of the relevant items (item score was 1 if correct and 0 if incorrect). First, the descriptive statistics for the full RAPM total score and the total sum score of all items belonging to each subset (i.e., subset total score) were retrieved and presented. Subsequently, for each subset, the total subset score was divided by the number of items within that subset to derive the subset mean score. Whereas the total score was used to report the descriptive results, the mean score was used to achieve norm subset scores when comparing the item hardness between two subsets and calculating gF-related correlations.

The decision to reply (in the present study) on DeShon and colleagues’ (1995) visuospatial and verbal-analytic subsets as the behavioral markers of visuospatial and verbal-analytic reasoning, respectively, is based on a rationale combining the following elements: (1) a preference for a simple, meaningful and (from a psychological perspective) well-interpretable structure, and (2) the avoidance of structures that may be the result of statistical artefacts. Our preference for a simple, meaningful and (from a psychological perspective) well-interpretable structure should clearly favor DeShon and colleagues’ (1995) pair of RAPM subsets over Carpenter and colleagues’ (1990) pair of RAPM subsets. Indeed, the RAPM subsets proposed by Carpenter *et al*. (1990) are mainly based on item properties and rules (e.g., addition or subtraction), not based on possible cognitive functions in reasoning (e.g., visuospatial reasoning) as is the case in DeShon and colleagues’ (1995) subsets. In fact, DeShon and colleagues’ (1995) RAPM subsets offer (just one pair of) distinct types of reasoning (i.e., visuospatial and verbal-analytic reasoning), a distinction which does have relevance from a psychological point of view.

Next, our aim to avoid structures that may be the result of statistical artefacts should also favor DeShon and colleagues’ RAPM subsets over Dillon and colleagues’ (1981) RAPM subsets. As Dillon and colleagues’ (1981) identified RAPM subsets mainly based on exploratory factor analysis, reliance on Dillon and colleagues’ (1981) RAPM subsets would imply a danger of: (1) simply labeling factors representing RAPM subsets based exclusively on statistical criteria, more specifically based on estimated factor loadings; and (2) encountering problems with the validity of key analytical outcomes (i.e., derived factors and thus also RAPM subsets) due to nonstandard (but realistic) analytical conditions, for instance the skewed (and thus ‘non-normal’) distributions of responses typically given to RAPM items; these nonstandard analytical conditions are known to make exploratory factor analysis imprecise (see Vigneau & Bors, 2005). Of note, a Kolmogorov-Smirnov test of (univariate) normality showed for all RAPM items a significant test result (all *ps* < 0.001), indicating that all RAPM items’ responses followed a non-normal distribution. Therefore, the application of exploratory factor analyses to RAPM items may lead to RAPM structures which (partly) reflect statistical artefacts^51,52^. As DeShon and colleagues (1995) did not derive RAPM subsets from factor-analytic results (on non-normal data), reliance on their RAPM subsets is not likely to be affected by statistical artefacts.

### Brain image acquisition and preprocessing

The rs-fMRI scan was performed on a 3-T scanner (Siemens Magnetom Trio, A Tim System) with a 12-channel phased-array head coil. Participants were instructed to rest without engaging in any specific task and remain still with their eyes closed and head motionless during the entire scan. The rs-fMRI scan lasted 8 minutes while participants were resting, and 240 contiguous echo planar imaging (EPI) volumes were produced (TR = 2000 ms; TE = 30 ms; flip angle = 90°; number of slices = 33; acquisition voxel size = 3.125 × 3.125 × 3.6 mm^3^). High-resolution T1-weighted images were also produced with magnetization prepared gradient echo sequence (MPRAGE: TR/TE/TI = 2530/ 3.39/ 1100 ms; flip angle = 7°; number of slices = 128; matrix = 256 × 256; voxel size = 1 × 1 × 1.33 mm^3^) for registration purposes.

rs-fMRI image preprocessing was performed with FSL (FMRIB’s Software Library, www.fmrib.ox.ac.uk/fsl)^53^. rs-fMRI image preprocessing included the following four steps: (1) removal of the first four brain volumes obtained for every participant, (2) head motion correction (by aligning [with MCFLIRT] each brain volume to the middle brain volume in the time domain of the rs-fMRI images), spatial smoothing (with a Gaussian kernel of 6 mm full-width half-maximum [FWHM]), intensity normalization, and removal of a possible linear trend in rs-fMRI’s time series of signal values, (3) reduction of low-frequency drifts and high-frequency noise by applying a temporal band-pass filter (0.01–0.1 Hz)^54,55^, and (4) further elimination of physiological noises by regressing out the first derivatives of nuisance signals of cerebrospinal fluid (CSF) and white matter (WM). The whole brain average rs-fMRI image signal and motion correction parameters were regressed out when predicting the residual rs-fMRI’s time series of signal values. Once this four-step sequence was completed, the 4-D residual rs-fMRI’s time series of signal values (1-D rs-fMRI time series of 3-D brain volumes) was obtained for FC analyses^56^.

For the spatial normalization of rs-fMRI images, the following three steps were completed: (1) registration of each participant’s rs-fMRI images to that participant’s structural MRI image; this registration was carried out with FLIRT producing a 6-degrees-of-freedom linear affine transformation matrix, (2) registration of each participant’s structural MRI images to the Montreal Neurological Institute (MNI) space; this registration was also accomplished with FLIRT producing a 12-degrees-of-freedom linear affine transformation matrix^57,58^, and (3) with the above-specified linear affine transformation matrixes, the rs-fMRI images were normalized to the MNI space. The voxel size was 2 × 2 × 2 mm^3^.

### Global brain connectivity

GBC values were computed using the Resting-State fMRI Data Analysis Toolkit (REST, http://rest.restfmri.net/)^59^. The strength of FC was assessed using Pearson’s correlation coefficient computed between the rs-fMRI time series of signal values of two grey matter (GM) voxels taken from all GM voxels. The expression “all GM voxels” should be interpreted as all brain voxels “within the priori defined GM mask” (i.e. the voxels in this GM mask have a higher probability of belonging to the MNI template’s GM than belonging to the MNI template’s WM and CSF [(GM > WM) ∩ (GM > CSF)])^60^. For each participant, GBC analyses were performed in three steps. First, for each GM voxel, the FCs between this GM voxel and the all other GM voxels were computed; in total, N-1 Pearson’s correlation coefficients were computed for each GM voxel (N refers to the number of GM voxels). Second, since the impact of global signal regression^61,62^, the total FC strength (raw GBC value) of each GM voxel was determined as the total sum of that particular GM voxel’s positive correlation coefficients (i.e., a threshold of *r* > 0 was set for FC edges, see below for global signal regression). In this way, our GBC value is similar to the concept of weighted degree centrality in graph theoretical analysis^26,63^. The raw GBC value was computed for all N GM voxels. By specifying each voxel’s raw GBC value as this voxel’s GM level, each participant’s raw GBC image was produced. Third, using a z-transform (i.e., for each voxel subtract the mean GBC value and divide by its SD as calculated across voxels within the GM mask), the raw GBC image was converted to a “z-score GBC image”, the image that was used in further analyses examining the relationships between the GBC value and the visuospatial, verbal–analytic subset scores^32,64,65^. Besides, to find the hub regions in the brain, which may also highly correlate with gF, the mean z-score GBC image across participants was calculated by averaging participants’ z-score GBC images.

The reasons that we just used the positive FCs to calculate the GBC was because the global signal regression, which is part of fMRI data preprocessing, may induce spurious negative FCs^61,62^. Hypothetically, two alternative situations may produce negative FCs. Situation 1 occurs if negative FCs were due to the analytics of global signal regression analysis (i.e., they are spurious); in this situation the negative FCs do not reflect the true neural substrates of cognitive processing^61,62^. In contrast, situation 2 occurs if negative FCs were due to (functional) negative correlations between GM voxels (that is, negative correlations reflecting these voxels’ asynchronous neural physiological activities); in this situation the negative FCs do reflect the true neural substrates of cognitive processing (i.e., they are not spurious). In both situations GBC values are affected; the negative FCs experience substantive impact from global signal regression^32,61,62^; so, one may have serious doubts about the usefulness of GBC values which are based on negative FCs. To ensure that the GBC values adequately reflect the brain’s physiological activities^32,66,67^, the correlation analyses between the GBC values and the RAPM subsets scores were based on positive FCs (i.e., *r* > 0). Besides, a ‘control analysis’ with GBC values which were exclusively based on negative FCs was also performed to calculate the correlation between RAPM score and negative FCs. This control analysis demonstrated that the correlation between the RAPM score and negative FCs revealed a spatially similar but (in sign/direction) opposite brain-behavioral correlation pattern to the pattern emerging from positive FCs. Thus, although negative FCs were affected by global signal regression, the negative FCs still reflect the neural substrates of cognitive processing, and they also pointed to the stability and validity of our main results based on positive FCs only (Figure S1 in the supplementary study materials).

### The relationship between GBC value and visuospatial/verbal–analytic reasoning

To present a complete picture of the relationship between RAPM-measured gF and global brain functional connectivity the correlations between the full RAPM total score (that is, not RAPM’s subset scores) and the GBC value were first computed (with sex, age and mean framewise displacement (FD)^68^ regressed out). Then to depict the relationships between the GBC value on the one hand, and visuospatial/verbal–analytic reasoning on the other hand, we computed the voxel-specific partial correlations between participants’ visuospatial or verbal-analytic subset scores and GBC value (with sex, age and mean FD as well as ‘the other subset score’ being regressed out). By relying on partial correlations we ensured that nuisances (e.g., due to sex, age and mean FD-differences) and the other subset score (i.e., the shared covariance) did not affect the size of the voxel-specific correlation coefficients computed for each RAPM subset. Besides, to also ensure that this residual regression model (i.e., the shared covariance of RAPM subsets was regressed out) did not distort the results that would be obtained based on just one subset, we also performed, for comparative purposes, “control analyses” with regression models in which “the other subset score” was not regressed out. Our control analyses showed that the correlational patterns are very consistent between the residual regression model on the one hand and the regression model based on just one of the two reasoning subsets on the other hand (Figures S2 and S3 in the supplementary study materials).

Furthermore, one has a risk to misinterpret the following kind of observation: certain regions show a specific significant brain-behavioral correlation for one reasoning type, but these regions’ corresponding brain-behavioral correlation is non-significant for the other reasoning type. One may then misinterpret this observation as an indication of the existence of significant distinct neural substrates between visuospatial and verbal–analytic subsets. To avoid such misinterpretation and further compare and confirm the functional discriminations between the neural substrates of visuospatial and verbal–analytic reasoning, we performed an interaction analysis between the two subsets’ items brain-behavioral correlations with the GBC value (i.e., the difference or “contrast” between the visuospatial and verbal–analytic regression slopes is determined). Because visuospatial reasoning relies more on visual perception operations and verbal-analytic reasoning relies more on logical operations^5,10,11^, a 6-mm (radius) spherical region of interest (ROI) was determined for visuospatial reasoning (i.e., visuospatial reasoning ROI) using the peak correlation coordinate in the primary visual cortex, and for verbal-analytic reasoning (i.e., verbal-reasoning ROI) using the peak correlation coordinate in the inferior frontal gyrus. The GBC values of these ROIs were extracted and the value differences between the visuospatial and verbal–analytic subsets were graphically depicted to inspect the nature of the brain-behavioral GBC interactions between these two RAPM subsets.

In line with previous studies on intelligence brain networks^69,70^, the correlations between RAPM scores and within- and between- network FCs of gF-related visual network and frontal-parietal multiple demand regions (MDr) were examined to further understand the distinct neural mechanisms underlying visuospatial and verbal-analytic reasoning. More specifically, with the visual network and MDr templates (i.e., the FPN, attention network [DAN] and ventral attention network [VAN] of Yeo and colleagues, 2011)^71^ and based on our interaction results (Fig. 5), we further explored the within- and between- network GBC (WNC/BNC GBC) of the PVC and IFG regions for visuospatial and verbal-analytic subsets^27,28^. Specifically, after calculating the WNC and BNC GBC of the visual network and MDr, we defined 6 mm spherical PVC and IFG ROIs according to their peak coordinate in Table 4 (i.e., 6, −76, 8 and 44, 28, 0), and then extracted WNC and BNC GBC values from these regions and correlated these GBC values with visuospatial and verbal-analytic subset scores to get two sets of correlations. The differences between these (sets of) correlations were examined with Z-tests.

Furthermore, to identify the core networks that contribute most to the visuospatial and verbal-analytic distinction in MDr, the network-wise brain-behavioral correlations were analyzed for the visuospatial and verbal-analytic subsets. First, the FPN, cingulo-opercular network (CON), SAN, DAN and VAN templates of Power and colleagues (2011) were contained to include the MDr (Fig. 7d)^72^. Second, the mean within-network connectivity (mWNC, e.g., the mean FCs between all FPN nodes) and mean between-network connectivity (mBNC, e.g., averaging all FCs between each couple of FPN and SAN nodes) were calculated to achieve the mean FCs within each network and between two networks. Third, the correlations between these network-wise FCs and RAPM subsets scores were calculated. Fourth, the differences between the visuospatial and verbal-analytic network-wise brain-behavioral correlations were examined using a Z-test (after a Fisher r-to-z transformation had been applied).

Significance testing of the cluster size (i.e., the number of contiguous voxels in a cluster) of these voxel-specific correlations relied on Monte Carlo simulation as included in AFNI’s AlphaSim program’s implementation of a multiple comparisons correction^73^. This correction relied on the following steps^74^: (1) The FWHM smoothness values of the brain-behavioral correlation image were computed using AFNI’s 3dFWHMx to estimate and then control the non-isotropic of smoothness^32,75^, (2) A Monte Carlo simulation to generate 1,000 simulated cluster sizes was conducted within the priori defined GM mask. The simulation parameters included the voxel level threshold of *p* < 0.01 (two-tailed) and the above computed FWHM in an orthonormal 3-D space. This step would create a distribution specifying 1,000 simulated cluster sizes. Consequently, all identified clusters containing more contiguous voxels than the cluster size of *p* < 0.05 in the distribution as determined in this second step were conceived as (multiple comparisons corrected) significant clusters.

In addition, to give the reader an insight into the stability of our results under different voxel-level thresholds, the above results were presented with a stricter voxel-level threshold of *p* < 0.001 and AlphaSim corrected threshold of *p* < 0.05 (Figure S4 and Table S1 in the supplementary study materials). Consistent with our original analyses the repeated analyses showed that the GBC of MFG, dACC, angular and supramarginal gyrus are correlated with the RAPM score. Also consistent with our original analysis separate neural substrates of visuospatial and verbal–analytic subset were found in the PVC and rIFG. In sum, our original results turned out to be stable.

## Electronic supplementary material

Supplementary Materials
