# Supplementary material for: Distinct neural substrates of visuospatial and verbal-analytic reasoning as assessed by Raven’s Advanced Progressive Matrices
Source: Sci Rep. 2017 Nov 24;7:16230. doi: 10.1038/s41598-017-16437-8 (PMC5701148; doi:10.1038/s41598-017-16437-8)
Supplement: Supplementary file 1 — Supplementary Materials [file 41598_2017_16437_MOESM1_ESM.doc]

**Supplementary control analyses and results**

related to the paper “**Distinct neural substrates of visuospatial and verbal-analytic reasoning as assessed by Raven’s Advanced Progressive Matrices**”

**Zhencai Chen, Alain De Beuckelaer, Xu Wang, Jia Liu**

**1. Control analysis pertaining to global signal regression**

When running the global signal regression (examining correlations between RAPM scores and GBC values) in the preprocessing phase some of the negative FCs that emerged may be artificial; that is, they do not reflect true neural correlates of cognitive processing, but analytical artefacts (Fox, Zhang, Snyder, & Raichle, 2009; Murphy, Birn, Handwerker, Jones, & Bandettini, 2009). Specifically, it has been demonstrated that global signal regression tends to shift the distribution of correlations downwards towards a mean correlation of zero (i.e., the “zero-centering” effect), and – as a consequence of this downward shift – create negative FCs in an artificial manner (Fox et al., 2009; Murphy et al., 2009).

To evaluate the impact of this downward shift (especially in terms of the inflated occurrence of negative FCs) when conducting the global signal regression, we performed a control analysis to test whether a specific (opposite) sign expectation (for a description: see just below) manifests itself in the data. Assuming that negative FCs are mainly caused by the “zero-centering” effect (i.e., the analytical artefact occurring in global signal regression), then the GBC values of positive FCs may have opposite direction (i.e., positive or negative) with those of negative FCs. If the sign is indeed opposite it is to be expected that the positive GBC image’s correlation with the RAPM score and the negative GBC image’s correlation with the RAPM score show similar brain-behavioral correlation regions, but the direction of the correlation (i.e., positive or negative) is opposite. To check this (opposite sign) expectation the correlation between the negative GBC images and the RAPM scores was calculated and presented in Figure S1. After contrasting Figure S1 to Figure 2 (in the paper), which represents the major brain-behavioral correlation regions of the positive GBC image, one must conclude that, indeed, the major brain-behavioral correlation regions of the positive GBC image and the major brain-behavioral correlation regions of the negative GBC image are similar but that the correlation goes in the opposite direction (i.e., positive versus negative). These correlational patterns observed also attested to the presence of a “zero-centering” effect occurring in global signal regression (Fox et al., 2009; Murphy et al., 2009), and its impact on the direction of the brain-behavioral correlation. As a consequence, in the present study it was decided to regress out the global signal and base the correlational analysis between GBC values and the RAPM score exclusively on positive correlations, a decision that was also made in previous studies (e.g., Michael W Cole, Ito, & Braver, 2015; M. W. Cole, Yarkoni, Repovs, Anticevic, & Braver, 2012).


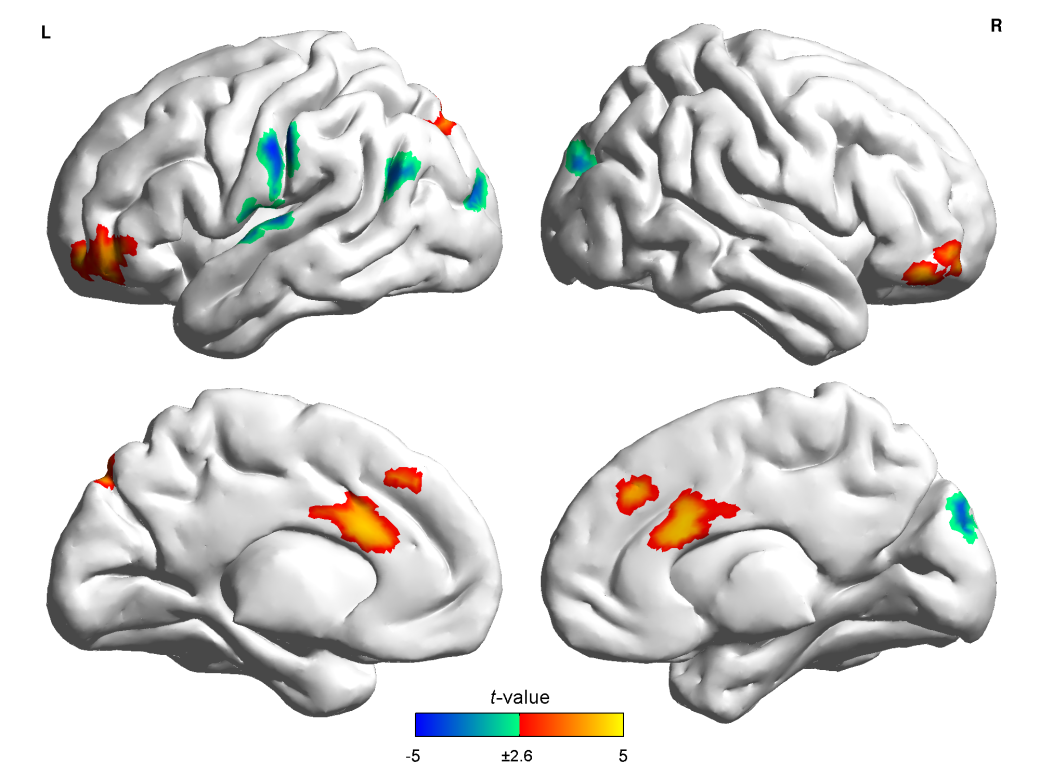


**Figure S1.** Regions with GBC values of negative FC showing significant correlations with the RAPM score after the effects of sex and age are regressed out. The correlational pattern is similar to the correlational pattern observed when relying on positive FCs only (see Figure 2 in the paper text).

**2. Control analyses pertaining to the residual regression model**

To ensure that results from our residual regression model (i.e., the regression model which regresses out the shared covariance between both RAPM subsets) did not deviate from results obtained by (the two) corresponding regression models involving only one RAPM subset, we also ran two additional regression models including only one RAPM subset’s score as the predictor of GBC values. In other words, the brain-behavioral correlations of visuospatial and verbal-analytic reasoning were determined separately.

The results from these two additional regression analyses are graphically depicted in Figures S2 and S3. Based on these figures on the one hand and the results of the residual regression model on the other hand (see Figures 3 and 4 in the paper) one must conclude that the correlational patterns as depicted for each type of reasoning’s subset are very consistent, that is stable across the residual regression model on the one hand and the two regression analysis models based on just one of the two types of reasoning’s subsets on the other hand. The stable correlational pattern indicates that the shared variance between both types of reasoning’s subsets does not really impact on the correlational patterns observed in our present study. In other words, the decision (made in the paper) to rely on the residual regression model is justifiable.


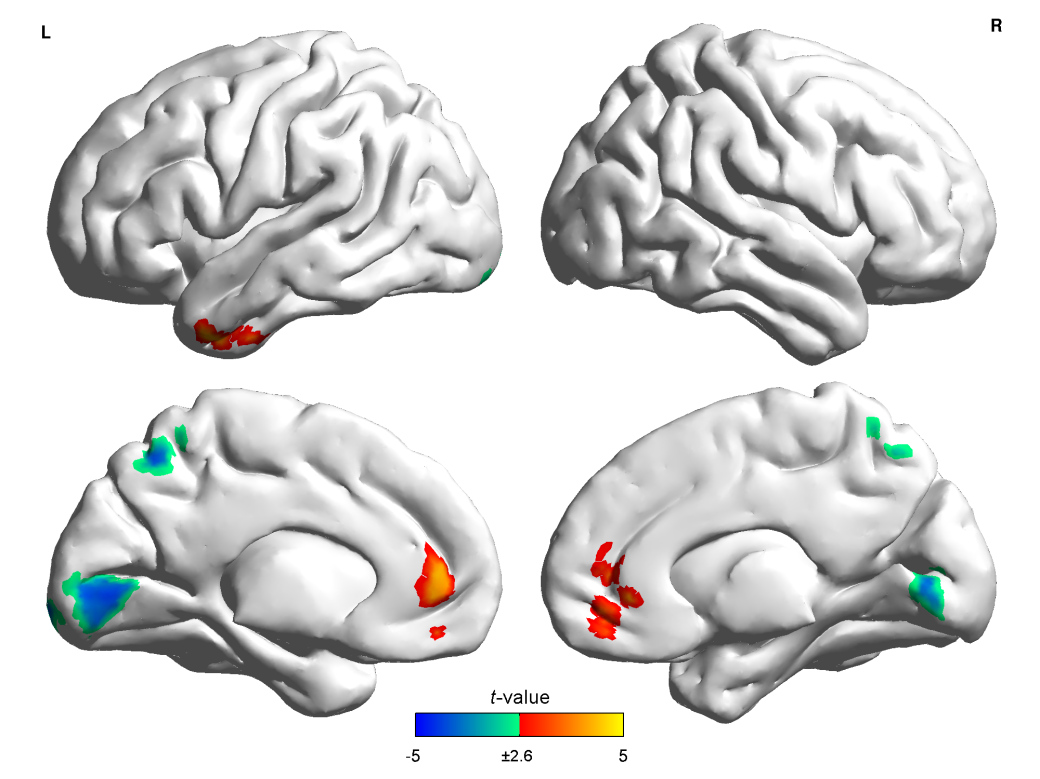


**Figure S2.** Regions with GBC values showing significant correlations with the visuospatial reasoning score after the effects of sex and age were regressed out. Note that the variance of the verbal-analytic subset score was not regressed out.


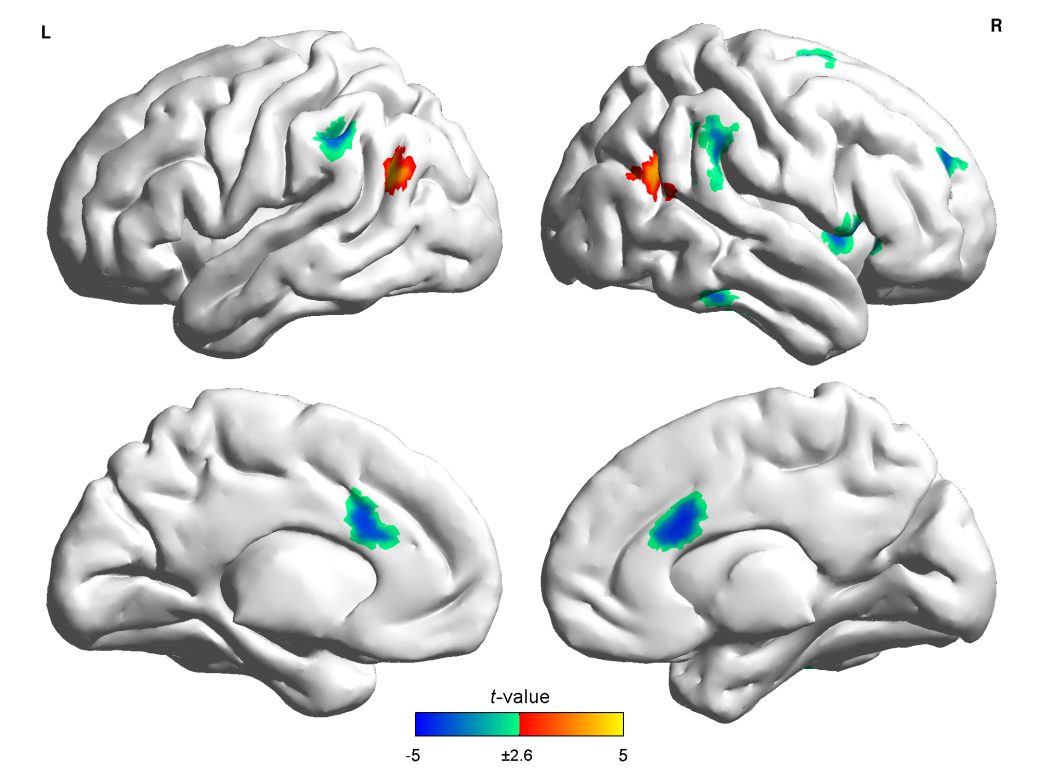


**Figure S3.** Regions with GBC values showing significant correlations with the verbal-analytic reasoning score after the effects of sex and age were regressed out. Note that the variance of the visuospatial subset score was not regressed out.

Moreover, correlation between both types of reasoning’s subsets was *r* = 0.43. Although such correlation is ‘moderate’, it does indicate that covariation between both types of reasoning’s subsets could only explain 18% of the total variation in each type of reasoning’s subset, leaving 82% of the total variation as variation that is unique to each particular type of reasoning’s subset. The high percentage (82%) demonstrates that both types of reasoning’s subsets are to be considered ‘independent’, at least as far as their impact on the correlational results is concerned. Since the present study focuses on the identification of separate neural mechanisms underlying the visuospatial and verbal-analytic subsets, a statistical removal of the covariation between both subsets (as implemented in our paper) is likely to be instrumental in adequately identifying the differences between visuospatial and verbal-analytic reasoning’s neural mechanisms.

**3. The AlphaSim corrected results (*p* < 0.05) at a voxel-level threshold of *p* < .001 supplementing the main results presented in the paper (see Figures 2 to 5) which relied on a voxel-level threshold of *p* < .01 (two-tailed).**

**
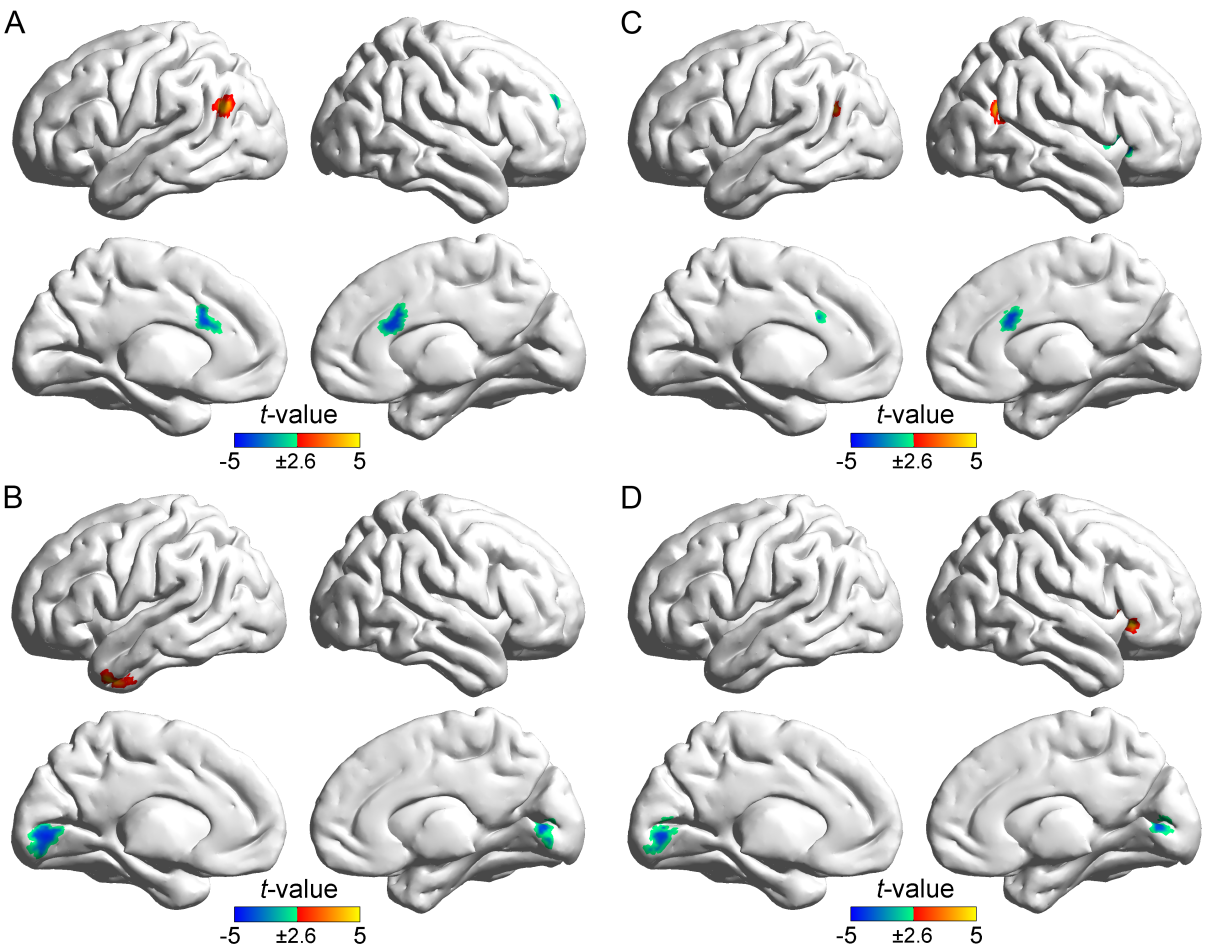
**

**Figure S4.** The main results at the voxel-level threshold of *p* < 0.001 and AlphaSim corrected at *p* < 0.05. (A) Regions with GBC values showing significant correlations with the RAPM score after the effects of sex and age scores were regressed out; (B) Correlational results between GBC values and the visuospatial subset score after regressing out the following covariates: sex, age, and the verbal–analytic subset score; (C) Correlational results between GBC values and the verbal–analytic subset score after regressing out the following covariates: sex, age, and the visuospatial subset score; (D) Regions showing the interaction between visuospatial and verbal-analytic reasoning. For each result, clusters containing more than 62 (A), 59 (B), 54 (C), and 59 (D) contiguous voxels survived the Alphasim correction.

| **Table S1**  Regions with GBC values showing significant correlations with the RAPM, the visuospatial and the verbal-analytic subset scores and the interaction between visuospatial and verbal-analytic reasoning, at a voxel-level threshold of *p* < .001 and AlphaSim corrected at *p* < 0.05 | | | | | | |  |
| --- | --- | --- | --- | --- | --- | --- | --- |
| Region | Brodmann area | No. of voxels | Peak *t* | x | y | z | |
| **Full set of RAPM** |  |  |  |  |  |  | |
| R. Middle/ Superior Frontal Gyrus | 10 | 71 | -4.22 | 32 | 50 | 22 | |
| Dorsal anterior cingulate cortex | 24 | 127 | -4.03 | 4 | 20 | 24 | |
| L. Angular/ Supramarginal gyrus | 39 | 96 | 3.79 | -54 | -60 | 26 | |
| **Visuospatial subset** |  |  |  |  |  |  | |
| L. Middle/ Inferior temporal gyrus | 21/20 | 74 | 4.56 | -46 | 8 | -38 | |
| Cuneus/ Lingual gyrus | 18/17 | 272 | -4.29 | 0 | -80 | 0 | |
| **Verbal-analytic subset** |  |  |  |  |  |  | |
| R. Inferior Frontal Gyrus | 47 | 138 | -4.78 | 44 | 28 | 0 | |
| Dorsal anterior cingulate cortex | 24 | 47 | -3.99 | 4 | 18 | 28 | |
| L. Angular gyrus | 39 | 49 | 4.19 | -46 | -60 | 24 | |
| R. Angular/ Supramarginal gyrus | 39 | 66 | 4.03 | 52 | -54 | 24 | |
| **Interaction between subsets** |  |  |  |  |  |  | |
| R. Inferior Frontal Gyrus | 47 | 109 | 4.88 | 44 | 28 | 0 | |
| Cuneus | 18 | 156 | -4.03 | 6 | -76 | 8 | |

**4. Interaction patterns as observed between visuospatial and verbal-analytic reasoning for the regions listed in the up panel of Figure 5 of the paper except for PVC and rIFG.**

**
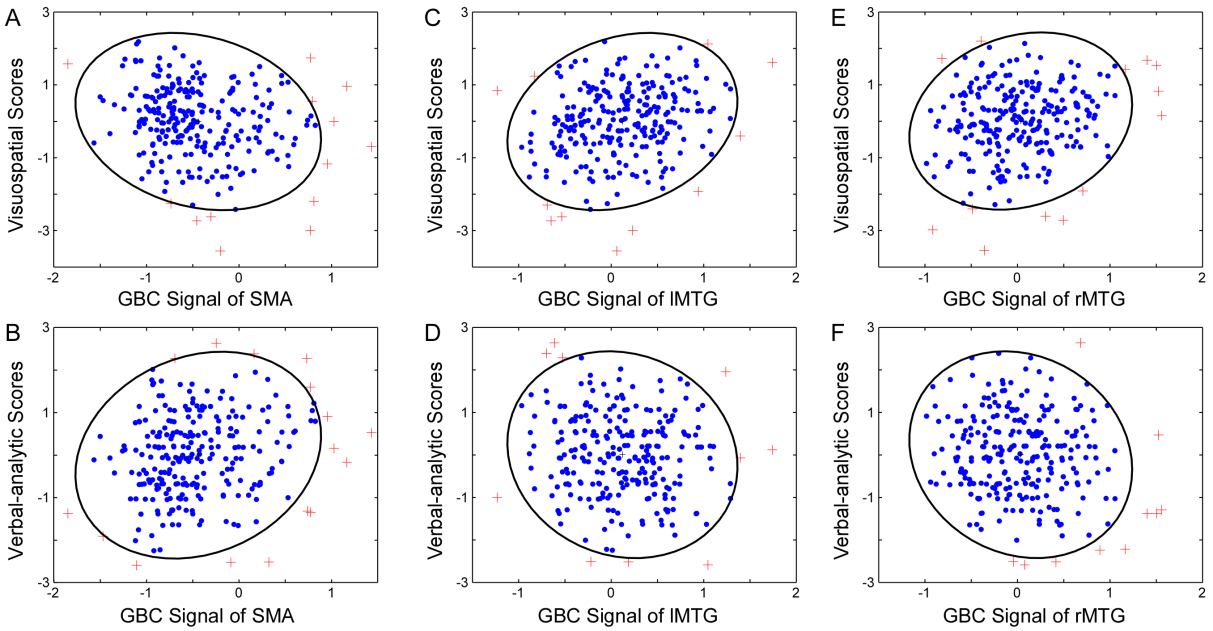
**

**Figure S5.** The brain-behavioral GBC interactions for visuospatial (top) and verbal-analytic reasoning (bottom) in the supplementary motor area (SMA, A/B), left middle temporal gyrus (lMTG, C/D) and right middle temporal gyrus (rMTG, E/F). The visuospatial and verbal-analytic reasoning scores were centered using a z-transform. The elliptic boundary defines the 95% confidence region.

**References**

Cole, M. W., Ito, T., & Braver, T. S. (2015). Lateral Prefrontal Cortex Contributes to Fluid Intelligence Through Multinetwork Connectivity. *Brain Connectivity, 5*, 497-504.

Cole, M. W., Yarkoni, T., Repovs, G., Anticevic, A., & Braver, T. S. (2012). Global connectivity of prefrontal cortex predicts cognitive control and intelligence. *J Neurosci, 32*, 8988-8999.

Fair, D. A., Dosenbach, N. U., Church, J. A., Cohen, A. L., Brahmbhatt, S., Miezin, F. M., et al. (2007). Development of distinct control networks through segregation and integration. Proceedings of the National Academy of Sciences, 104(33), 13507-13512.

Fox, M. D., Zhang, D., Snyder, A. Z., & Raichle, M. E. (2009). The global signal and observed anticorrelated resting state brain networks. *J Neurophysiol, 101*, 3270-3283.

Murphy, K., Birn, R. M., Handwerker, D. A., Jones, T. B., & Bandettini, P. A. (2009). The impact of global signal regression on resting state correlations: are anti-correlated networks introduced? *NeuroImage, 44*, 893-905.

Rubinov, M., & Sporns, O. (2010). Complex network measures of brain connectivity: uses and interpretations. NeuroImage, 52(3), 1059-1069.

Wang, X., Song, Y., Zhen, Z., & Liu, J. (2016). Functional integration of the posterior superior temporal sulcus correlates with facial expression recognition. Human brain mapping.

Wang, X., Zhen, Z., Song, Y., Huang, L., Kong, X., & Liu, J. (2016). The Hierarchical Structure of the Face Network Revealed by Its Functional Connectivity Pattern. The Journal of Neuroscience, 36(3), 890-900.
